# Febuxostat attenuates aluminum chloride-induced hepatorenal injury in rats with the impact of Nrf2, Crat, Car3, and MNK-mediated apoptosis

**DOI:** 10.1007/s11356-023-28182-9

**Published:** 2023-06-20

**Authors:** Ahmed A. Sedik, Soha A. Hassan, Heba I. Shafey, Wagdy K. B. Khalil, Noha A. Mowaad

**Affiliations:** 1 Pharmacology Department, Medical Research and Clinical Studies Institute, National Research Center, El-Buhouth St., Dokki, Cairo, 12622 Egypt; 2 Basic Science Department, Faculty of Dentistry, October 6 University, Giza, Egypt; 3 Department of Cell Biology, National Research Centre, El-Buhouth St., Dokki, Cairo, 12622 Egypt; 4 Narcotics, Ergogenics and Poisons Department, Medical Research and Clinical Studies Institute, National Research Center, El-Buhouth St., Dokki, Cairo, 12622 Egypt

**Keywords:** Aluminum, Febuxostat, Nrf2, Crat, car3, COX-1, Caspase-3, Hepatorenal injury

## Abstract

Aluminum (Al) is a ubiquitous xenobiotic with known toxicity for both humans and animals. Our study was conducted to investigate the protective role of febuxostat (Feb) against aluminum chloride (AlCl3)-induced hepatorenal injury in rats. Hepatorenal injury was induced by oral administration of AlCl3 (40 mg/kg b.w.), for 2 months. Twenty-four male Sprague–Dawley rats were randomly allocated into four groups (six rats/group). The first group received the vehicle thought the experiment. The second group was considered as a control positive group. The third and fourth groups received oral treatment of Feb (10 mg/kg.b.w.) and (15 mg/kg.b.w.), respectively with AlCl3, concurrently for 2 months. Twenty-four hours, after the last treatment, serum biochemical, molecular, histopathology, and immunohistochemical studies were evaluated. Our findings showed that rats intoxicated with Alcl_3_ had disturbed biochemical picture. In addition, intoxication with AlCl3 increased oxidative stress and apoptosis, as demonstrated by an increase in malodialdeyde (MDA), carnitine o-acetyltransferase (Crat), and carbonic anhydrase (Car3) with a decrease in glutathione (GSH), MAP kinase-interacting serine/threonine kinase (MNK) and nuclear factor-erythroid 2-related factor 2 (Nrf2) mRNA expression. Furthermore, the levels of tumor necrosis factor-alpha (TNF-α) and the levels of caspase-3 were elevated with sever hepatic and renal pathological changes. Conversely, Feb (15 mg/kg.b.w.) could improve the serum biochemical indices and repressed MDA, Crat, and Car3 levels, whereas it increased GSH, MNK, and Nrf2 levels. Feb inhibited the apoptotic effect of AlCl3 in the liver and kidney by decreasing caspase-3 and TNF-α expression. The protective effect of Feb against AlCl3 toxicity was confirmed by histopathological findings. Moreover, molecular docking studies supported the anti-inflammatory effect of Feb due to its significant binding interactions with cyclooxygenase-1 (COX-1), NF-kappa-B-inducing kinase (NIK), and mitogen-activated protein kinases-p38 (MAPK-p38). The findings suggest that Feb system Feb can avert Alcl3-induced hepatotoxicity and nephrotoxicity by enhancing the antioxidant defense system, and inhibiting the inflammatory cascade and apoptosis.

## Introduction

Aluminum (Al) is one of the most abundant metallic elements on the planet. The availability of AL has recently drawn more attention to its biotoxicity (Al Eisa and Al Nahari 2016; Machado-Neves et al. 2023). AL is used as a food additive, in cooking pots with roughly 20% Aluminum content, in drinking water with a 0.2-mg/L concentration, as a water-purifying agent, in can bottles, in aluminum foil paper, and in antiperspirant cosmetic products. Although having a low gastrointestinal absorption capacity (less than 1%), it may accumulate over time in vital organs like the kidney, liver, and brain, where it may cause apparent neurotoxicity and cytotoxicity (Othman et al. 2020).

Additionally, AL may stimulate the pro-oxidant features of iron and copper, which results in mitochondrial dysfunction, the oxidative degradation of macromolecules, and the release of cytochrome C from the mitochondria (Al-Kahtani et al. 2020). Moreover, AL can induce the pro-oxidant features of iron and copper, resulting in mitochondrial dysfunction, oxidative degradation of macromolecules, and the release of cytochrome C from mitochondria and apoptosis. Therefore, eliminating AL toxicity through neutralization and scavenging of free radicals may be a viable option.

A selective xanthine oxidase inhibitor called febuxostat (Feb) is used to treat hyperuricemic conditions including gout and tumor lysis syndrome by lowering urate level (Pui et al. 2012). Due to their potential benefits in the treatment of several autoimmune and inflammatory illnesses, xanthine oxidase inhibitors have come under more attention. These inhibitors have anti-inflammatory, antioxidant, and immune-modulatory properties. Various experimental models had shown anti-inflammatory properties of febuxostat (Fahmi et al. 2016). In the rat model of renal ischemia–reperfusion injury, it inhibited the formation of ROS and had an antioxidative stress effect (Tsuda et al. 2012).

Determining if Feb has any potential protective benefits against Alcl3-induced hepatorenal damage in rats was the goal of the current study. Additionally, potential pathways behind in Feb-mediated effects were investigated.

## Materials and methods

### Experimental animals

Twenty-four adult male Sprague–Dawley rats (150–170 g) about 6 weeks old were housed in separated metal cages at the animal house, October 6 University, Giza, Egypt. Rats were housed in a well-ventilated room, under ambient laboratory conditions (22 ± 1 °C temperature, 45–55% relative humidity) with a 12-h light/12-h dark cycle. Water and food were freely available. Under approval number (RECO6U/10–2022), our study was carried out under the ethical standards documented by the Research Ethics Committee, Faculty of Dentistry, October 6 University, Giza, Egypt.

### Drugs and chemicals

Aluminum Chloride (Alcl3; CAS number: 7446–70-0) and febuxostat (Feb; CAS number: 144060–53-7) were obtained from Sigma-Aldrich Co. (USA). All other chemicals and kits were of highest analytical grade. The LD_50_ of oral administration of Alcl3 was reported previously at 400 mg/kg b.w. (Yousef 2004). While Feb was regarded safe in rats at (15 mg/kg/day) (Takeda Pharmaceuticals America 2009).

### Experimental design

Rats were divided blindly into four groups of six each, as follows: normal control group: rats were received the vehicle thought the experiment. Alcl3 group: rats were received orally Alcl3 (40 mg/kg b.w.) for 2 months (Okail et al. 2020). Alcl3 + Feb10 group: rat received oral treatment of febuxostat (10 mg/kg.b.w.) concurrently with Alcl3 for 2 months (Hwang et al. 2014; Tsuda et al. 2012). Alcl3 + Feb 15 group: rat received oral gavage treatment of febuxostat (15 mg/kg.b.w.) concurrently with Alcl3 for 2 months.

At the end of the experiment, about 2 ml blood sample were drawn from the eye’s retro-orbital plexus of the rats after being anesthetized with ketamine (100 mg/kg) and xylazine (10 mg/kg) and centrifuged at 1500 rpm for 10 min for separation of serum (− 20 °C) for monitoring serum hepatic enzymes, total lipids, total cholesterol, triglycerides, total protein and albumin levels (El-Baz et al. 2020).

Rats were decapitated under light anesthesia, and their liver and kidney were immediately excised; and dived into two parts: part 1 being kept at − 80 °C for conducting biochemical and molecular analysis, while part 2 was fixed in 10% neutral formalin for evaluating the histopathogical and immunohistochemical alterations in liver and kidney.

#### Preparation of liver and kidney homogenate

The preserved liver and kidney from each rat were perfused with cold phosphate-buffered saline (PBS; pH 7.4) after being cleaned in regular saline solution and blotted over filter paper. Then, an automatic tissue homogenizer (Heidolph, Germany) was used to homogenize 1 g of tissues in 9 volumes of cold PBS (pH 7.4). The tissue homogenate was centrifuged at – 80 °C and 4000 rpm for 15 min, and the supernatant was kept at − 80 °C for evaluation of reduced glutathione (GSH) and malondialdehyde (MDA).

### Evaluation of serum biochemical indices

Alanine aminotransferase (ALT) and aspartate aminotransferase (AST) were measured colorimetrically at 546 nm (Reitman and Frankel 1957). Alkaline phosphatase (ALP) was assayed colorimetrically at 520 nm (Belfield and Goldberg 1971). Total lipids, total cholesterol and triglycerides concentrations were estimated (Zollner and Kirsch 1962), (Richmond 1973), (Fossati and Prencipe 1982). Total protein and albumin levels were evaluated (Lowry 1951), (Doumas et al. 1971).

### Evaluation of hepatic and renal levels of GSH (nmol/g tissue) and MDA (nmol/g tissue)

The activity of GSH in the liver and kidney tissues was assayed colorimetrically (Beutler 1963). Levels of lipid peroxidation (LPO) in the liver and kidney tissue were determined by measuring MDA levels (Ohkawa et al. 1979).

### Evaluation of oxidative stress and cell related genes

#### Isolation of total RNA

The standard TRIzol® Reagent extraction method was used to isolate total RNA from liver and kidney tissue samples. Tissue samples were homogenized in a mortar using liquid nitrogen and 1 ml of TRIzol® Reagent. Prior to use, RNA was dissolved in diethylpyrocarbonate (DEPC)-treated water (Salem et al. 2018).

#### Reverse transcription (RT) reaction

Utilizing the RevertAidTM First Strand cDNA Synthesis Kit, the complete hepatic and renal samples from Poly (A) + RNA were reversely transcribed into cDNA (Table 1).

**Table 1:** Primers sequence used for *qRT-PCR*

Gene	Primers sequence	NCBI reference
Nrf2	F: TCT TAC GCA TTC CCC AAC CT	XM_039105942.1
	R: CGA CCC GCT TAA TGA CAA GG	
MNK	F: CAC AGG GAC CTA AAG CCA GA	NM_001011985.2
	R: GCT TGT CAT AGA TGC TGG CC	
Crat	F: AAG AAG CCT GAA CTT GTG CG	NM_001004085.2
	R: TTA GCT TCT GGG ACT TGG GG	
Car3	F: GGC GAG TTC CAG ATT CTC CT	NM_019292.4
	R: CTG GTC TGA GCT CAC TGT CA	
GAPDH	F: AAC GAC CCC TTC ATT GAC CT	DQ403053.1
	R: CCC CAT TTG ATG TTA GCG GG	

*(Nrf2)* nuclear factor erythroid 2–related factor 2, *MNK* MAP kinase-interacting serine/threonine kinase, *Crat* carnitine O-acetyltransferase, *Car3* carbonic anhydrase 3, *GAPDH* glyceraldehyde-3-phosphate dehydrogenase, *F* forward, *R* reverse

#### Real-time-polymerase chain reaction (RT-PCR)

The copy number of the rat tissues samples was ascertained using Thermo Fisher Scientific's StepOneTM Real-Time PCR System from Applied Biosystems. A melting curve analysis was carried out at 95.0 °C after of each qPCR to evaluate the effectiveness of the primers used (Table 1) (Khalil and Booles 2011).

### Comet assay

Comet assay was scored in liver and kidney tissues samples from different experimental groups (Blasiak et al. 2004). The slides were stained with ethidium bromide and observed under a × 400 Zeiss epifluorescence microscope (510–560 nm, barrier filter 590 nm, 100 cell/rat). Randomly chosen non-overlapping cells were scored visually on a scale of 0–3 based on perceived comet tail length migration and relative proportion of DNA in the nucleus (class 0 = no detectable DNA damage and no tail; class 1 = tail less than the diameter of the nucleus; class 2 = tail between 1 and 2 the diameter of the nucleus; and class 3 = tail longer than 2 the diameter of the nucleus) (Collins et al. 1997; Olive et al. 1990).

### Histopathological examination of hepatic and renal tissues

Liver and kidney tissues were diced and fixed in formaldehyde solution (10%) at room temperature for 48 then washed, dehydrated in alcohol, cleared in xylol, and finally embedded in paraffin wax blocks. Sections (5 μm) were cut using Leica microtome, mounted, and stained as usual with hematoxylin and eosin (Gamble et al. 2008).

### Imuunohistochemical evaluation of hepatic and renal levels of caspase-3 and TNF-α

Sections were stained by stained by the IHC technique according to data sheet. TNF-α Rabbit pAb (A11534) antibody from ABclonal (catalog no. A11534; 500 W Cummings Park, Ste. 6500 Woburn, MA 01801, USA). Anti-cleaved-Caspase-3 Rabbit pAb antibody from servicebio (catalog no.GB11009: East Lake High-Tech Developing Zone, Wuhan, Hubei, China 430,079). In brief, mounted sections were deparaffinized and incubated with 3% H2O2 solution for 15 min to rinse slides with PBS and treated Incubated with primary antibody overnight at 4 °C. After rinsing in PBS peroxidase-labeled secondary antibodies were applied and incubated for 30 min. Counterstaining was done using hematoxylin (Sedik and Hassan 2022; Pedrycz and Czerny 2008).

### Evaluation of pharmacodynamic, pharmacokinetics parameters and insilico toxicity of febuxostat

The pharmacokinetic parameters of Feb were evaluated by the Swiss target prediction software (http://www.swisstargetprediction.ch/). Physicochemical properties, lipophilicity, aqueous solubility, absorption, and pharmacokinetic parameters were evaluated.Glory software was also used to determine the potential metabolic route and metabolites of Feb (https://nerdd.zbh.uni-hamburg.de/glory/) (de Bruyn Kops et al. 2019). Additionally, CLC-Pred software was used to assess the toxicity of Feb against a variety of cell lines (http://www.way2drug.com/Cell-line/).

### Evaluation of molecular docking results of febuxostat

Molecular docking of Feb against, cyclooxygenase-1 (COX-1, PDB ID; 6Y3C-A), NF-kappa-B-inducing kinase (NIK, PDB ID; 4DN5-A), and mitogen-activated protein kinases-p38 (MAPK-p38, PDB ID; 1A9U-A) were performed after retrieval of three-dimensional protein structures from RCSB-PDB database (https://www.rcsb.org/). Protein structures were prepared with by BIOVIA Discovery Studio (Vélizy-Villacoublay, France). Therefore, we selected the A chain of 6KDL, 4A69, and 3V2A for protein preparation by removal of water molecules and all ligands in addition to energy minimization and refinement processes. In addition, the 3D structure of Feb was obtained from the PubChem database (https://pubchem.ncbi.nlm.nih.gov/). The binding free energy, binding affinity (p*Ki*), and the ligand efficiency of Feb against prepared proteins were determined using InstaDock software (Hassan et al. 2017). Finally, BIOVIA Discovery Studio Visualizer software did the visualization of target-ligand interaction.

### Statistical analysis

One-way analysis of variance (ANOVA, GraphPad Prism program 8.0, USA) and general liner models (GLM) procedure of Statistical Analysis System (1982) were used for all quantifiable comparisons in our study. Tukey’s multiple comparison and Scheffé tests were utilized to score the significance. Results are presented as mean ± SEM of six rats and the difference was documented significant when *p* value is ≤ 0.05.

## Results

### Effect of febuxostat on serum hepatic markers in rats intoxicated with Alcl3

**Fig. 1:**
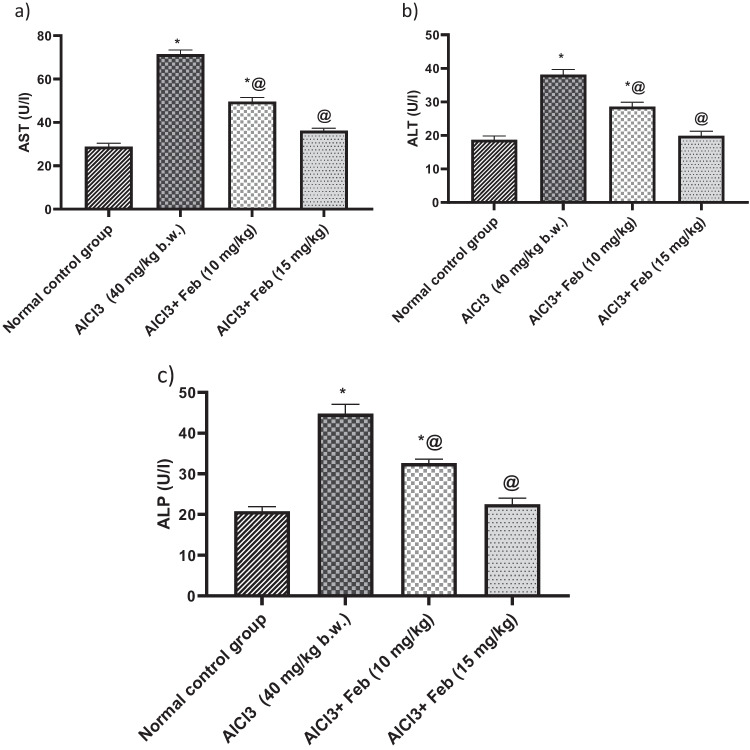
Effect of febuxostat on serum hepatic markers in rats intoxicated with Alcl_3_. The hepatorenal injury was induced in rats via oral administration of Alcl_3_ (40 mg/kg. b.w.) for 2 months. Intoxicated rats with Alcl_3_ were orally treated with two doses of Feb (10 mg/kg. b.w.) and (15 mg/kg. b.w.) concurrently with Alcl_3_ for 8 weeks. 24 h after the last treatment, levels of AST, ALT and ALP were evaluated. *Significant difference from normal control group p < 0.05. @ Significant difference from intoxicated rats with Alcl_3_

Alcl3-induced hepatorenal injury showed a highly significant elevation (*p* < 0.05) by 250%, 200%, and 215% in the values of AST, ALT, and ALP, respectively, as compared with normal control group. Treatment with Feb 10 mg/kg, significantly (*p* < 0.05) decreased AST, ALT and ALP by 69%, 75%, and 72% respectively, as compared to Alcl3 treated group. While, treatment with Feb 15 mg/kg could restore the aforementioned parameters to their normal values (Fig. 1).

### Effect of febuxstat on urea, creatinine, and uric acid levels in rats intoxicated with Alcl3

**Fig. 2:**
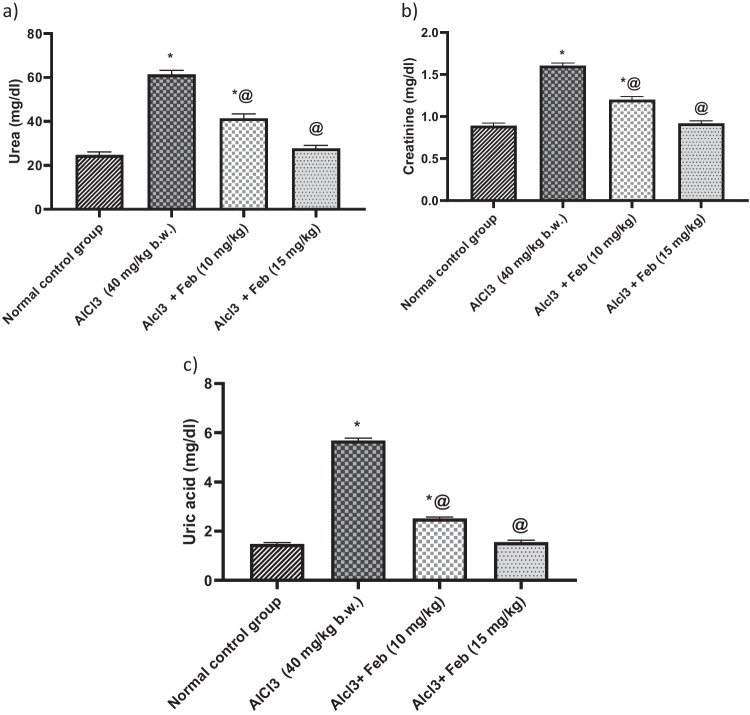
Effect of febuxostat on urea, creatinine and uric acid levels in rats intoxicated with Alcl_3_. The hepatorenal injury was induced in rats via oral administration of Alcl_3_ (40 mg/kg. b.w.) for 2 months. Intoxicated rats with Alcl_3_ were orally treated with two doses of Feb (10 mg/kg. b.w.) and (15 mg/kg. b.w.) concurrently with Alcl_3_ for 8 weeks. 24 h after the last treatment, levels of urea, creatinine and uric acid were evaluated. *Significant difference from normal control group p < 0.05. @ Significant difference from intoxicated rats with Alcl_3_

Administration of Alcl3 for 2 months was associated with increased levels of urea, creatinine and uric acid (*p* < 0.05) by 248%, 180%, and 3.5-folds, as compared with a normal control group. Treatment with Feb 10 mg/kg, significantly (*p* < 0.05) decreased urea, creatinine, and uric acid by 67%, 75%, and 44% respectively, as compared to Alcl3-treated group. While, treatment with Feb 15 mg/kg could restore the a formentioned parameters to their normal values (Fig. 2).

### Effect of febuxstat on albumin and total protein levels in rats intoxicated with Alcl3

**Fig.3:**
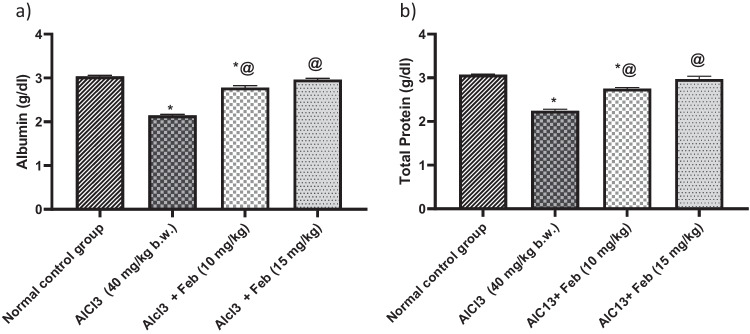
Effect of febuxostat on albumin and total protein levels in rats intoxicated with Alcl_3_. The hepatorenal injury was induced in rats via oral administration of Alcl_3_ (40 mg/kg. b.w.) for 2 months. Intoxicated rats with Alcl_3_ were orally treated with two doses of Feb (10 mg/kg. b.w.) and (15 mg/kg. b.w.) concurrently with Alcl_3_ for 8 weeks. 24 h after the last treatment, levels of albumin and total protein were evaluated. *Significant difference from normal control group p < 0.05. @ Significant difference from intoxicated rats with Alcl_3_

Administration of Alcl3 for 2 months was associated with decreased levels of albumin and T. protein (*p* < 0.05) by 79% and 72%, as compared with normal control group. Treatment with Feb 10 mg/kg, significantly (*p* < 0.05) increased albumin and T. protein by 129% and 122% respectively, as compared to Alcl3-treated group. While treatment with Feb15 mg/kg could restore the aforementioned parameters to their normal values (Fig. 3).

### Effect of febuxstat on triglycerides, total cholesterol, and total lipids levels in rats intoxicated with Alcl3

**Fig.4:**
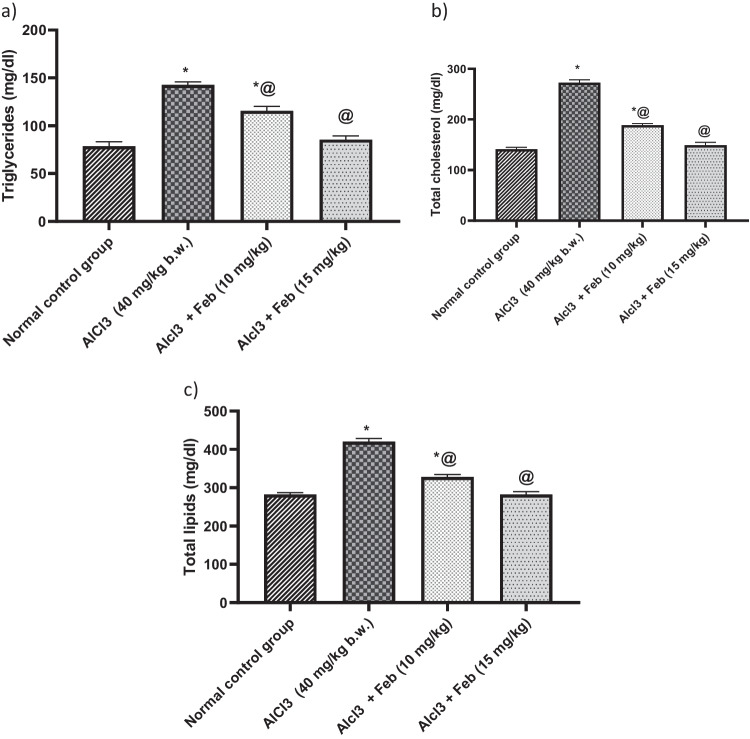
Effect of febuxostat on triglycerides, total cholesterol and total lipids levels in rats intoxicated with Alcl_3_. The hepatorenal injury was induced in rats via oral administration of Alcl_3_ (40 mg/kg. b.w.) for 2 months. Intoxicated rats with Alcl_3_ were orally treated with two doses of Feb (10 mg/kg. b.w.) and (15 mg/kg. b.w.) concurrently with Alcl_3_ for 8 weeks. 24 h after the last treatment, levels of triglycerides, total cholesterol and total lipids were evaluated. *Significant difference from normal control group p < 0.05. @ Significant difference from intoxicated rats with Alcl_3_

Administration of Alcl3 for 2 months was associated with increased levels of triglycerides, totalcholesterol, and total lipids (*p* < 0.05) by 181%, 193%, and 148%, as compared with normal control group. Treatment with Feb 10 mg/kg, significantly (*p* < 0.05) decreased triglycerides, total cholesterol and total lipids by 81%, 69%, and 79% respectively, as compared to Alcl3 treated group. While treatment with Feb 15 mg/kg could restore aforementioned parameters to their normal values (Fig. 4).

### Effect of febuxstat on the hepatic and renal levels of GSH and MDA in rats intoxicated with Alcl3

Hepatic and renal levels of GSH were significantly (*p* < 0.05) decreased in Alcl3- exposed group by 36% and 43%, respectively, as compared to the control group. Treatment with Feb 10 mg/kg, significantly (*p* < 0.05) increased the hepatic and renal levels of GSH by 2.5-folds and 163% respectively, as compared to Alcl3 treated group. While treatment with Feb 15 mg/kg could normalize the hepatic and renal levels of GSH (Table 2).

**Table 2:** Effect of febuxostat on the hepatic and renal values of GSH and MDA in rats intoxicated with Alcl_3_

Treatment	GSH (nmol/g tissue)		MDA (nmol/g tissue)	
	Hepatic	Renal	Hepatic	Renal
Normal control group	39.02 ± 0.15	27.82 ± 0.4	22 ± 1.2	62.33 ± 1.5
Alcl_3_ group (40 mg/kg)	14.19 ± 0.3^*^	11.99 ± 0.7^*^	89 ± 3.8^*^	149.7 ± 1.4^*^
Alcl_3_ + Feb (10 mg/kg) group	35.26 ± 0.5^*@^	19.66 ± 0.8*@	45.8 ± 2.1	84.67 ± 1.8^*@^
Alcl_3_ + Feb (15 mg/kg) group	37.83 ± 0.4^@^	25.44 ± 0.8^@^	27.3 ± 1.8^@^	69 ± 2.1^@^

The hepatorenal injury was induced in rats via oral administration of Alcl_3_ (40 mg/kg. b.w.) for 2 months. Intoxicated rats with Alcl_3_ were orally treated with two doses of Feb (10 mg/kg. b.w.) and (15 mg/kg. b.w.) concurrently with Alcl_3_ for 8 weeks. 24 h after the last treatment, hepatic and renal levels of GSH and MDA were evaluated

^*^Significant difference from normal control group *p* < 0.05

@Significant difference from intoxicated rats with Alcl_3_

Hepatic and renal levels of MDA were significantly (*p* < 0.05) increased in Alcl3-exposed group by 4-folds and 3-folds, respectively, as compared to the control group. Treatment with Feb 10 mg/kg, significantly (*p* < 0.05) decreased the hepatic and renal levels of MDA by 62% and 56%, respectively, as compared to Alcl3-treated group. While treatment with Feb15 mg/kg could normalize the hepatic and renal levels of MDA (Table 2).

### Effect of febuxstat on the expression of Nrf2, MNK, Crat, and Car3 genes in hepatic and kidney tissues in rats intoxicated with Alcl3.

**Fig.5:**
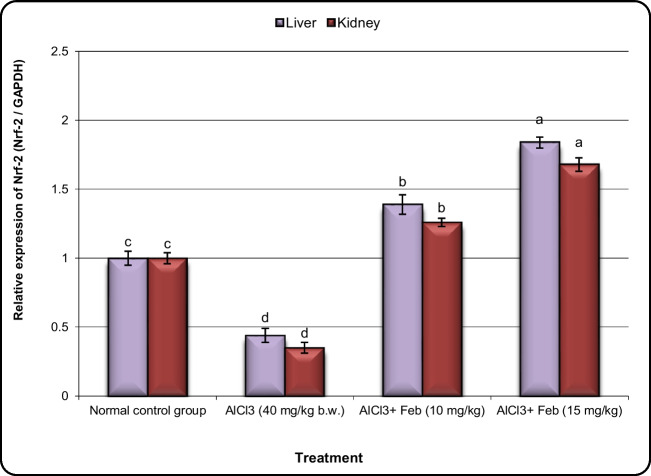
Effect of febuxostat on the expression alterations of Nrf-2 gene in the liver and kidney in rats intoxicated with Alcl_3_. The hepatorenal injury was induced in rats via oral administration of Alcl_3_ (40 mg/kg. b.w.) for 2 months. Intoxicated rats with Alcl_3_ were orally treated with two doses of febuoxstat (10 mg/kg. b.w.) and (15 mg/kg. b.w.) concurrently with Alcl_3_ for 8 weeks. 24 h after the last treatment, the expression alterations of Nrf-2 gene in liver and kidney samples were evaluated. Data are presented as mean ± SD. Means with different superscripts (^a,b,c^) between groups in the same column are significantly different at P < 0.05

**Fig.6:**
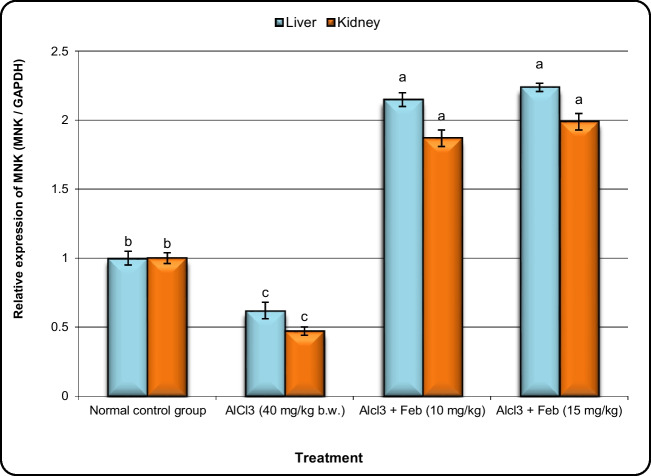
Effect of febuxostat on the expression alterations of MNK gene in the liver and kidney in rats intoxicated with Alcl_3_. The hepatorenal injury was induced in rats via oral administration of Alcl_3_ (40 mg/kg. b.w.) for 2 months. Intoxicated rats with Alcl_3_ were orally treated with two doses of Feb (10 mg/kg. b.w.) and (15 mg/kg. b.w.) concurrently with Alcl_3_ for 8 weeks. 24 h after the last treatment, the expression alterations of MNK gene in liver and kidney samples were evaluated. Data are presented as mean ± SD. Means with different superscripts (^a,b,c^) between groups in the same column are significantly different at P < 0.05

**Fig.7:**
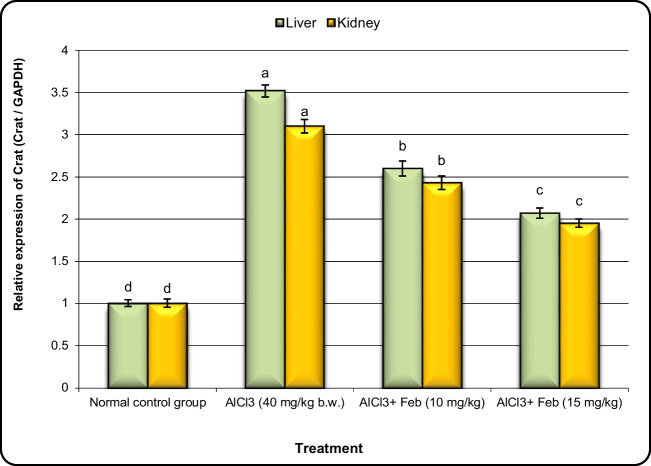
Effect of febuxostat on the expression alterations of Crat gene in the liver and kidney in rats intoxicated with Alcl_3_. The hepatorenal injury was induced in rats via oral administration of Alcl_3_ (40 mg/kg. b.w.) for 2 months. Intoxicated rats with Alcl_3_ were orally treated with two doses of Feb (10 mg/kg. b.w.) and (15 mg/kg. b.w.) concurrently with Alcl_3_ for 8 weeks. 24 h after the last treatment, the expression alterations of Crat gene in liver and kidney samples were evaluated. Data are presented as mean ± SD. Means with different superscripts (^a,b,c,d^) between groups in the same column are significantly different at P < 0.05

**Fig.8:**
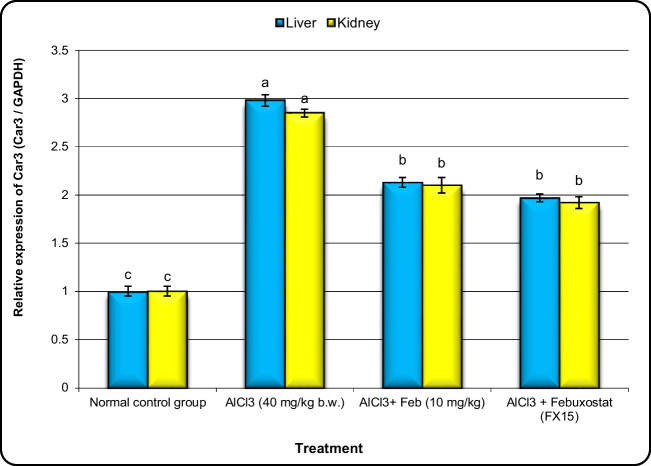
Effect of febuxostat on the expression levels of Car3 gene in the liver and kidney in rats intoxicated with Alcl_3_. The hepatorenal injury was induced in rats via oral administration of Alcl_3_ (40 mg/kg. b.w.) for 2 months. Intoxicated rats with Alcl_3_ were orally treated with two doses of Feb (10 mg/kg. b.w.) and (15 mg/kg. b.w.) concurrently with Alcl_3_ for 8 weeks. 24 h after the last treatment, the expression alterations of Car3 gene in liver and kidney samples were evaluated. Data are presented as mean ± SD. Means with different superscripts (^a,b,c^) between groups in the same column are significantly different at P < 0.05. Data are presented as mean ± SD. Means with different superscripts (^a,b,c,d^) between groups in the same column are significantly different at P < 0.05

The expression levels of oxidative stress (Nrf2), cell cycle (MNK), and initial carcinogenesis (Crat and Car3) related genes in liver and kidney tissues are shown in Figs. 5, 6, 7, and 8, respectively. The Nrf2 and MNK expression levels in liver and kidney tissues were down-regulated significantly (***P*** < 0.05) in the group of rats exposed to Alcl3 compared with control rats (Figs. 5 and 6). However, the expression levels of Nrf2 and MNK in the liver and kidney tissues of Alcl3-exposed rats treated with Feb (10 mg/kg) were increased significantly in comparison to those in rats exposed to Alcl3 only. Additionally, expression levels in liver and kidney tissues of Alcl3-exposed rats treated with Feb (15 mg/kg) were increased much more than those in Alcl3-exposed rats treated with Feb (10 mg/kg) regarding Nrf2 gene but not the case in MNK gene.

The expression levels of Crat and Car3 genes in liver and kidney tissues were upregulated significantly (*P* < 0.01) in the group of rats exposed to Alcl3 compared with control rats (Figs. 7 and 8). However, the expression levels of Crat and Car3 in the liver and kidney tissues of Alcl3-exposed rats treated with Feb (10 mg/kg) were decreased significantly in comparison to those in rats exposed to Alcl3 only. Additionally, expression levels of Crat and Car3 in liver and kidney tissues of Alcl3-exposed rats treated with Feb (15 mg/kg) declined much more than those in Alcl3-exposed rats treated with Feb* (*10 mg/kg).

### Effect of febuxstat on the rate of DNA damage in hepatic and kidney tissues in rats intoxicated with Alcl3

The rate of DNA damage in liver and kidney tissues of rats exposed to Alcl3 and treated with Feb is summarized in Tables 3 and 4, respectively.

**Table 3:** Visual score of DNA damage in liver tissues of exposed to AlCl3 and treated with febuxostat

Treatment		No. of cells		Class^**^				DNA damaged cells % (Mean ± SEM)
	No of samples	Analyzed^*^	Comets	0	1	2	3	
Normal control group	6	600	44	556	35	9	0	7.33 ± 0.49^c^
Alcl_3_ group (40 mg/kg)	6	600	127	473	41	39	47	21.17 ± 1.14^a^
Alcl_3_ + Feb (10 mg/kg) group	6	600	95	505	32	38	25	15.83 ± 0.95^b^
Alcl_3_ + Feb (15 mg/kg) group	6	600	87	513	34	31	22	14.50 ± 0.76^b^

^*^Number of cells examined per a group

^**^Class *0* = no tail,* 1* = tail length < diameter of nucleus, *2* = tail length between 1 and 2X the diameter of nucleus; and *3* = tail length > 2X the diameter of nucleus. Data are presented as mean ± SEM

^a,^^b,c,d^Mean values within tissue with unlike superscript letters were significantly different (*P* < 0.05)

**Table 4:** Visual score of DNA damage in kidney tissues of exposed to AlCl3 and treated with febuxostat

Treatment		No. of cells		Class^**^				DNA damaged cells % (Mean ± SEM)
	No of samples	Analyzed^*^	Comets	0	1	2	3	
Normal control group	6	600	43	557	37	6	0	7.18 ± 0.71^c^
Alcl_3_ group (40 mg/kg)	6	600	118	482	44	40	34	19.67 ± 0.88^a^
Alcl_3_ + Feb (10 mg/kg) group	6	600	89	511	36	32	21	14.84 ± 1.17^b^
Alcl_3_ + Feb (10 mg/kg) group	6	600	81	519	38	29	14	13.52 ± 0.78^b^

^*^Number of cells examined per a group

^**^Class *0* = no tail, *1* = tail length < diameter of nucleus,* 2* = tail length between 1 and 2X the diameter of nucleus; and *3* = tail length > 2X the diameter of nucleus. Data are presented as mean ± SEM

^a,^^b,c,d^Mean values within tissue with unlike superscript letters were significantly different (*P* < 0.05)

The rates of DNA damage in the group of rats exposed to Alcl3 were increased significantly (***P*** < 0.01) compared with that in control rats (Tables 1 and 3). However, the DNA damage in the liver and kidney tissues of Alcl3-exposed rats treated with Feb (10 mg/kg) were decreased significantly in comparison to those in rats exposed to Alcl3 only. Moreover, the DNA damage in the liver and kidney tissues of Alcl3-exposed rats treated with Feb (15 mg/kg) were reduced much more than those in Alcl3-exposed rats treated with Feb (10 mg/kg) but without significant differences.

### Effect of febuxstat on the histopathological picture in the liver and kidney in rats intoxicated with Alcl3

**Fig. 9:**
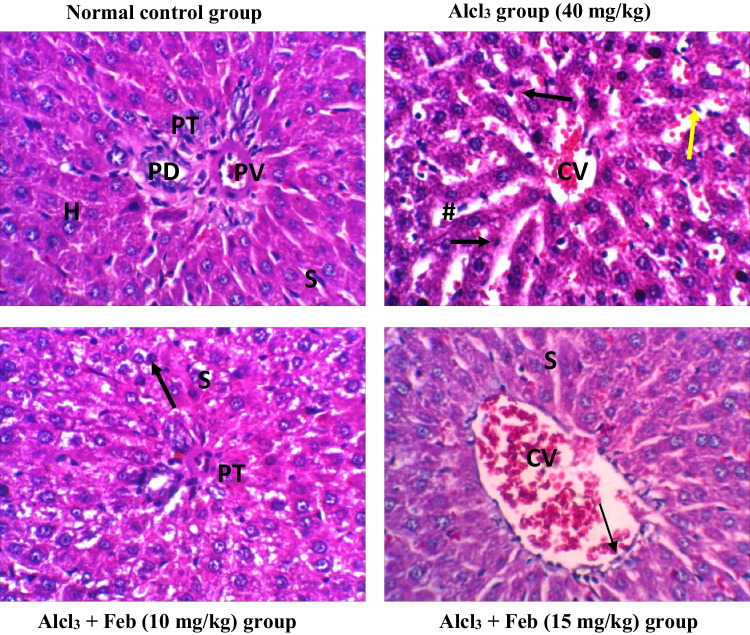
photomicrographs of H&E staining rat liver sections of control and different experimental groups. Normal control group showing normal liver tissue histology with normal hepatic cell aggregation. Hepatocyte (H) appears in usual shape rows separated by hepatic sinusoids (S), normal hepatic portal tract (PT) with the normal portal vein (PV) and bile duct (PD). AlCl_3_ group (40 mg/kg) showing complete loss of normal hepatic cord architecture with necrotic. Degeneration and necrotic hepatocyte (black arrow) around congested central veins (CV) and inflammatory cell aggregation (Yellow arrow) and swollen sinusoids (#). Alcl_3_ + Feb (10 mg/kg) group showing mild shrunken portal tract (PT), mild coagulative necrosis of some hepatic cells (black arrow) slightly swelled hepatic sinusoids (S). Alcl_3_ + Feb (15 mg/kg) group showing most hepatic cord preserve the normal histological appearance, with congestion central vein (CV) lined with normal endothelial cells (thin arrow) and normal sinusoids (S), with an increase in Kupffer cells (Kc) for regeneration. Magnification X: 400

**Fig. 10:**
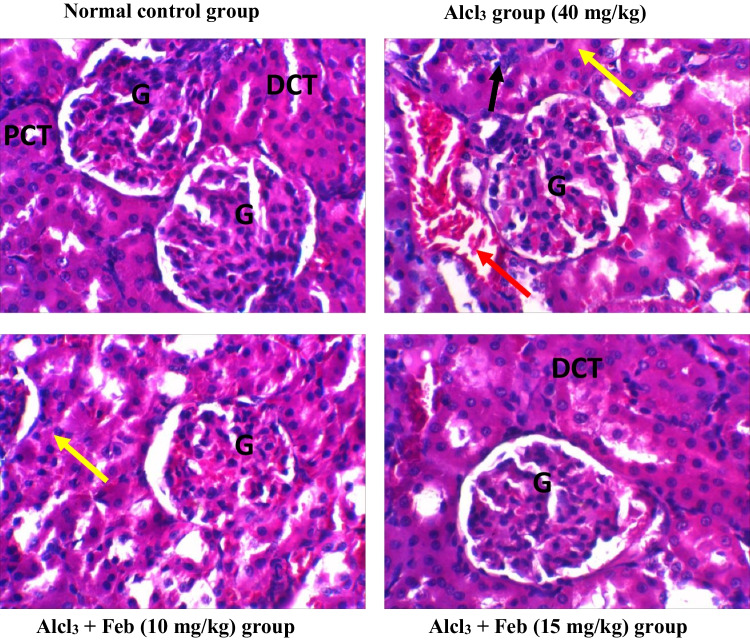
photomicrographs of H&E staining rat kidney sections of control and different experimental groups. Normal control group showed a normal structure of the kidney with glomerulus (G), proximal convoluted tubule (PCT) and distal convoluted (DCT). Alcl_3_ group (40 mg/kg) showed severe congestion and hemorrhage of glomerular tuft (G), swelling of renal tubules (yellow arrow) with renal casts in lumen (black arrow), Edema in the interstitial tissue with lymphocytic cells infiltration around glomeruli and renal tubules (red arrows). Alcl_3_ + Feb (10 mg/kg) group showed mild congestion of glomerular tuft (G) and some tubules suffered cloudy swelling (yellow arrow). Alcl_3_ + Feb (15 mg/kg) group showed improvement in the kidney histology architecture resembling the control group with still little swelling cells and mild hemorrhage. Magnification X: 400

Alcl3-induced severe pathological alterations, marked loss of architecture, severe inflammatory cells were observed in the liver and kidney tissue. However, Alcl_3_ + Feb (15 mg/kg) group showed mild inflammation with few coagulative necrosis in some hepatic cells and nephrocytes. While Alcl_3_ + Feb (15 mg/kg) group showed normal histological appearance in liver and kidney tissues with an increase in Kupffer cells for regeneration and little hemorrhage and congestion in kidney tissue (Figs. 9 and 10).

### Effect of febuxstat on the expression levels of Caspase-3 in the liver and kidney in rats intoxicated with Alcl3

**Fig. 11:**
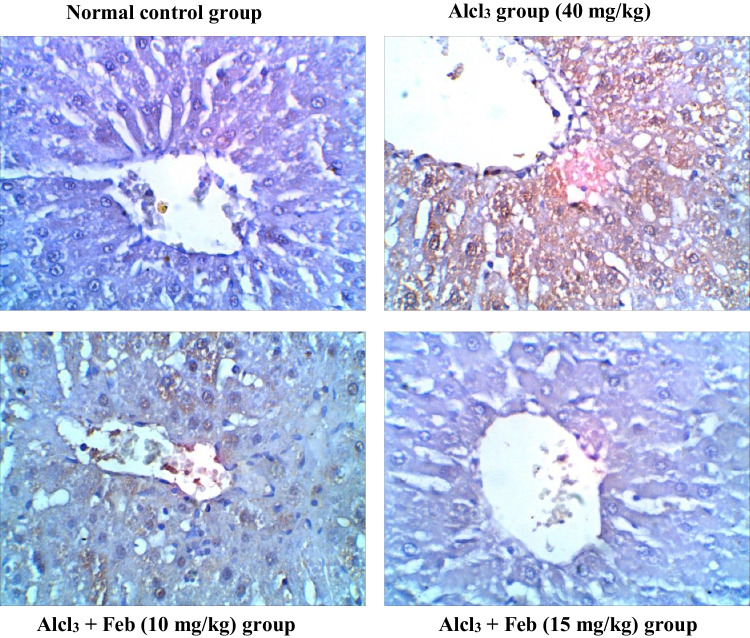
Immunohistochemical staining of Caspase-3 in liver sections from control and experimental groups. Normal control group revealing rare immunolabeled hepatic cells of Caspase -3. Alcl_3_ group (40 mg/kg) revealed brown staining with a slight increase in the number of Caspase-3 immunolabeled hepatocytes (that were observed around central veins suggesting inflammatory response and slight increase in apoptosis. Alcl_3_ + Feb (10 mg/kg) group showing slight decrease in Caspase-3 immunolabeled cells, indicating a slight improvement in the liver section. Alcl_3_ + Feb (15 mg/kg) group showed slight decrease in Caspase-3 immunolabeled cells, nearly normal to the control group

**Fig. 12:**
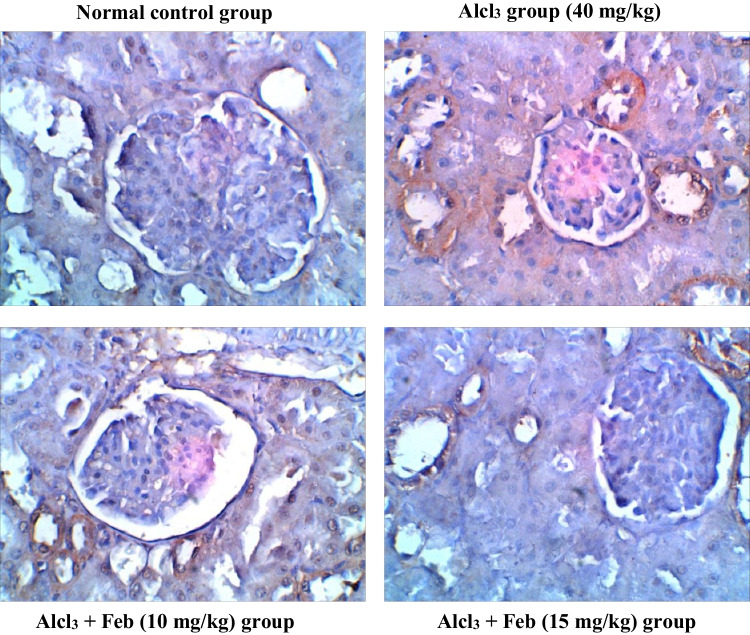
Immunohistochemical staining of Caspase-3 in kidney sections from control and experimental groups. Normal control group revealing negligible caspase-3 immunopositivity in the cortical regions of the kidney, glomerulus not stained brown. Alcl_3_ group (40 mg/kg) revealed strong increase in Caspase-3 expression in cortical areas especially in the proximal convoluted tubules as an inflammatory marker, whereas in the glomerulus was less Caspase-3 expression. Alcl_3_ + Feb (10 mg/kg) group showing partial inhibition in Caspase-3 expression indicating weak immune staining in distal convoluted tubules of cortical regions. Alcl_3_ + Feb (10 mg/kg) group showing noticed few cells were positive expression for cleaved Caspase-3 almost normal if compared to a normal control group

Oral administration of theAlcl3 group (40 mg/kg) for 2 months revealed marked increase in the number of hepatic and renal Caspase-3 immunolabeled cells around central veins suggesting inflammatory response and apoptosis. Alcl_3_ + Feb (10 mg/kg) group showing a slight decrease in hepatic and renal Caspase-3 immunolabeled cells, indicate a slight improvement in the liver and kidney section. Alcl_3_ + Feb (15 mg/kg) the group showed a significant reduction in Caspase-3 immunolabeled cells, nearly normal to control group (Figs. 11 and 12).

### Effect of febuxstat on the expression levels of TNF-α in the liver and kidney in rats intoxicated with Alcl3

**Fig. 13:**
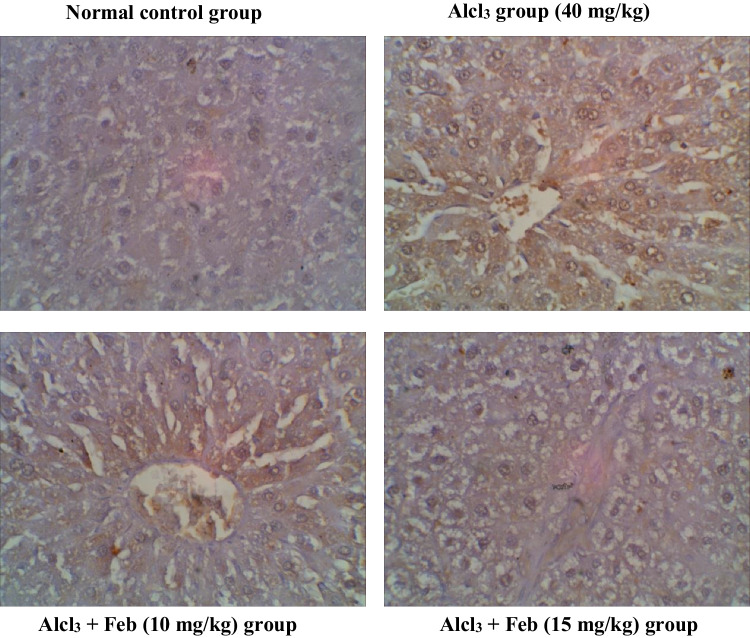
Immunohistochemical staining of TNF—α in liver sections from control and experimental groups. The normal control group revealed no expression of TNF-α immunostained hepatic cells. Alcl_3_ group (40 mg/kg) revealed a strong increase in TNF-α expression of apoptotic hepatocytes indicating hepatic injury. Alcl_3_ + Feb (10 mg/kg) group showing reduced expression of TNF-α of hepatocytes. Alcl_3_ + Feb (10 mg/kg) group showed a marked reduction in the expression of TNF-α in the liver section showing somewhat normal cells. Brown color indicates immunopositivity for each stain, CV: central vein. H: hepatocyte. Magnification: 40X

**Fig. 14:**
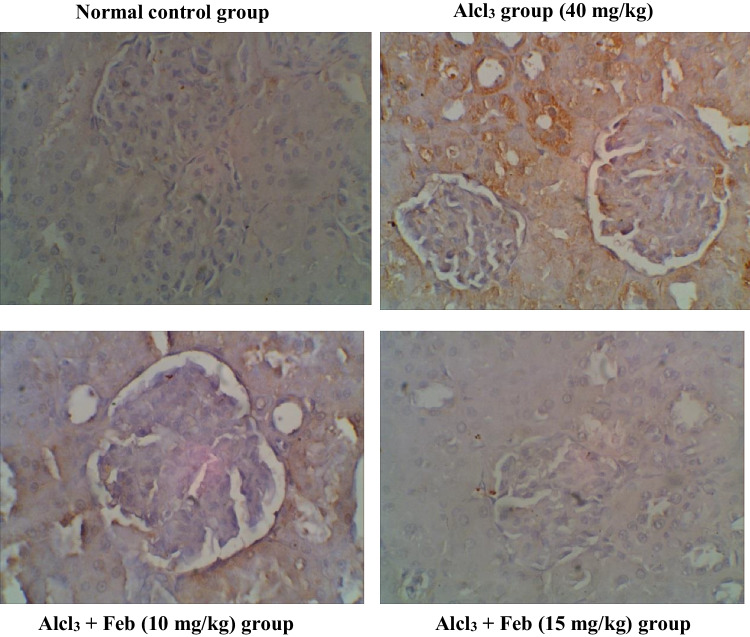
Immunohistochemical staining of TNF—α in kidney sections from control and experimental groups. The normal control group revealed no TNF-α positive stained cells in the cortical region of kidney tissue. Alcl_3_ group (40 mg/kg) showed that glomerular sections have high TNF-positive immunostaining indicating mononuclear inflammatory cells as starting the necrosis process. Alcl_3_ + Feb (10 mg/kg) group showing moderate TNF-α immune-reactive renal tubular cells as amelioration in kidney tissue. Alcl_3_ + Feb (10 mg/kg) group showing very weak TNF-α immune-reactive cells nearly normal if compared to control group staining. Brown color indicates immunopositivity for each stain, G: Glomerulus. CT: proximal convoluted tubules. Magnification: 40X

Oral administration of the Alcl3 group (40 mg/kg) for 2 months revealed marked increase in the number of hepatic and renal TNF-α, indicating mononuclear inflammatory cells to starting the necrosis process. Alcl_3_ + Feb (10 mg/kg) the group showed a slight decrease in hepatic and renal TNF-α, indicating a slight improvement in liver and kidney section. Alcl_3_ + Feb (15 mg/kg) the group showed a significant reduction in TNF-α, nearly normal to control group (Figs. 13 and 14).

### Results of pharmacokinetic of febuoxstat by Swiss target prediction software

**Fig. 15:**
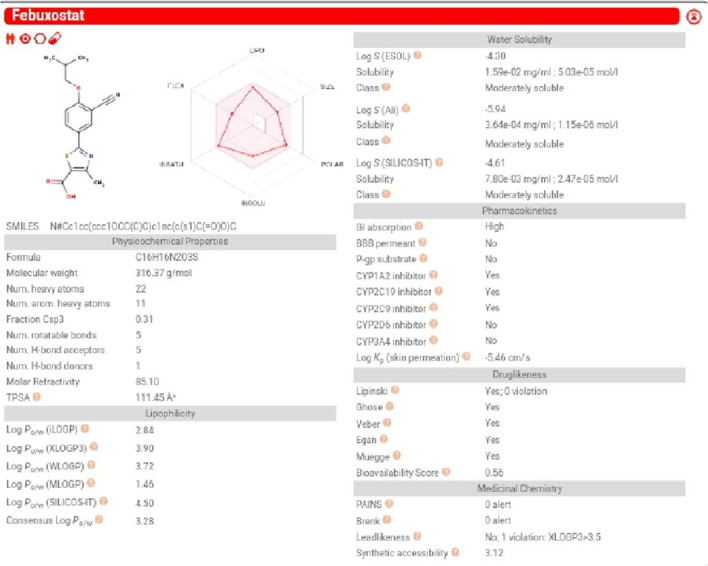
Evaluation of the physic-chemical and pharmacokinetic properties of febuxostat using Swiss target prediction software. The physicochemical properties that were evaluated are absorption, distribution, bioavailability and water solubility. Febuoxostat followed Lipinski, Ghose, Veber, Egan and Mueggr rules, while bioavailability scores, 0.56

**Fig. 16:**
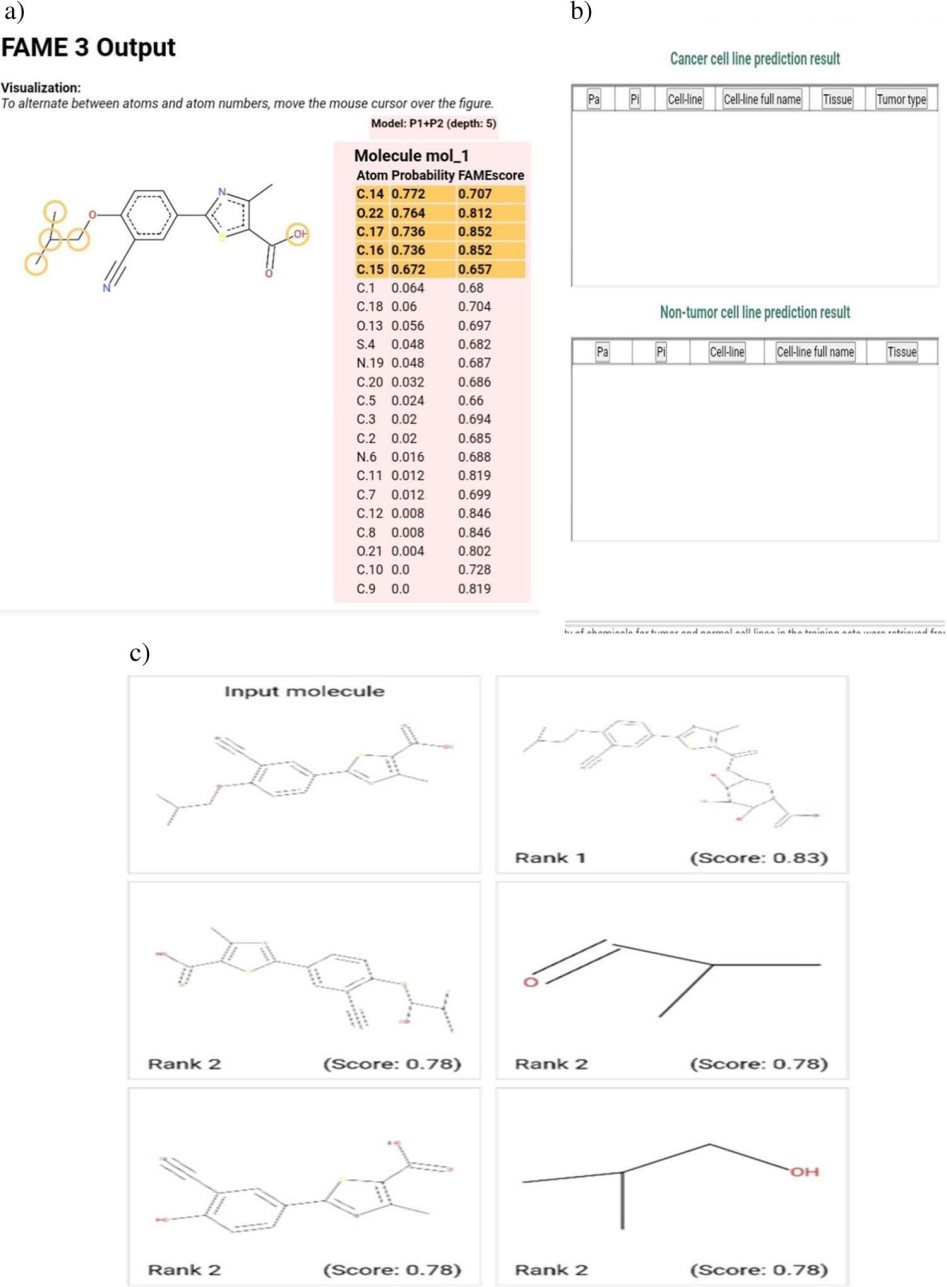
Evaluation of FAME1, possible metabolites and toxicity against cell lines. Swiss target prediction software was applied to score FAME1, products of phase 1 metabolism and toxicity against cell lines

Feb exhibits high GIT absorption, moderate water solubility, and low BBB permeability. Additionally, the Feb adhered to most drug-likeness rules, including the Lipinski rule. Feb showed no toxicity against the various cell lines (Figs. 15 and 16).

### Results of molecular docking of febuxstat

Molecular docking scores and interactions of Feb against target proteins are represented in Table 5 and Figs. 1, 2, and 3. Regarding cyclooxygenase-1 (COX-1), Feb made four hydrophobic (ARG83, PRO86 (2), and LYS473), and three hydrogen (SER85, ARG467, and GLU520) bonds during its binding with the COX-1 binding site (Fig. 1). Feb bound with the amino acid residues in the NF-kappa-B-inducing kinase (NIK) binding site by p*Ki* value of 5.43 (Table 5 and Fig. 2). Feb bound and interacted with NIK binding site by two charge (ARG363 (2)), six hydrophobic (ARG363, ARG366, ARG368, PRO372 (2), and LEU433), and one hydrogen (ARG363).

**Table 5:** Molecular docking results of febuxostat against NF-kappa-B-inducing kinase, MAPK-P38, and Cyclooxygenase-1 (COX-1)

Febuoxostat	Protein		Binding free energy (kcal/mol)	p*Ki*	Ligand efficiency (kcal/mol/non-H atom)
NF-kappa-B-inducing kinase (NIK) PDB ID; 4dn5_A		Scores	− 7.4	5.43	0.3364
		Interactions	Charge: ARG363 (2) Hydrophobic: ARG363, ARG366, ARG368, PRO372 (2), and LEU433 Hydrogen: ARG363		
MAPK-P38 PDB ID; 1a9u_A		Scores	− 6.2	4.55	0.2818
		Interactions	Hydrophobic: VAL30, TYR35, VAL38, and ARG173 Hydrogen: TYR35		
Cyclooxygenase-1 (COX-1) PDB ID; 6Y3C-A		Scores	− 6.6	4.84	0.3
		Interactions	Hydrophobic: ARG83, PRO86 (2), and LYS473 Hydrogen: SER85, ARG467, and GLU520		

Molecular docking was utilized to illustrate the interaction between febuxostat against NIK, MAPK-P38, and COX1. Binding free energy, PKi, and ligand efficiency were scored. In addition, the number and type of chemical interactions were detected

Results in Table 5 and Fig. 3 showed the p*Ki* values of Feb is (4.55) towardMAPK-p38. Feb interacted with MAPK-p38 binding site interacted by four hydrophobic (VAL30, TYR35, VAL38, and ARG173) and one hydrogen (TYR35).

Feb bound and interacted with the binding site of interleukin-1 beta convertase by two charge (ARG161 and ARD163) and four hydrophobic (TRP145, ILE155 (2), and LEU196) bonds with p*Ki* value of 3.81.

## Discussion

Aluminum (AL) is widely spread in the crust of the earth; *t* makes up about 8% of all the mineral elements. It is easy to enter our body because it is a component of cooking utensils and medications including anti-acids, deodorants, and food additives so toxicity of aluminum is considered due to its availability (Hassan and Kadry 2021b). Because of its great availability, AL can accumulate in the liver and kidney, making humans especially sensitive to its toxicity (Al Dera 2016). These findings suggest that the potential role of febuxostat (Feb) against Alcl3 toxicity-induced hepatorenal injury in rats. Febuxostat is available in two dose levels (10 and 15 mg/kg/day), with the later dose appearing to be the most efficient.

Based on our research, we can state with certainty that Feb has the greatest ability to scavenge ROS, activate antioxidant enzymes, and block the inflammatory cascade and apoptosis in order to prevent Alcl3-induced hepatotoxicity and nephrotoxicity.

Hence, Feb is essential for the creation of novel therapeutic strategies intended to combat Alcl3-induced hepatotoxicity and nephrotoxicity. Our findings showed that, when compared to the control group, the Alcl3-exposed group had significantly (*p* 0.05) lower levels of GSH and MDA in the liver and kidneys, When compared to the Alcl3-treated group, treatment with Feb 10 mg/kg significantly (*p* 0.05) enhanced the levels of GSH and MDA in the liver and kidneys. Although a treatment with Feb15 mg/kg could restore normal GSH and MDA levels to the liver and kidneys.

Feb administration to intoxicated rats could improve the antioxidant status and ameliorated the changes in biochemical indices and histopathological picture. Al is not a redox active metal, but it can produce reactive oxygen species (ROS). Aqueous solution of AlCl3 which administered orally for three months with a 10 mg/kg/day can lead to oxidative stress and cell damage in a variety of tissues, including the liver and kidney (Zatta et al. 2002).

Lipid peroxidation (MDA) is regarded as the primary indicator of oxidative damage in the toxicity of many xenobiotics (Mężyńska et al. 2019). As a result of increased free radical production, which reacts with polyunsaturated fatty acids to produce hydroperoxides and start a chain reaction that damages cell membranes by oxidation. Due to Feb’s potent antioxidant properties, administration of 15 mg/kg of Feb was successful in bringing down the elevated levels of MDA. This is in line with earlier research showing powerful antioxidant effects of Feb in a dose febuxostat (10 mg/kg; p.o.) for 21 days (Khames et al. 2017). Additionally, it has been demonstrated that Feb lowers ROS and MDA levels in the aortas of streptozocin-diabetic rats and atherosclerotic mice which administrate Feb 0.027 mg/mL with drinking water for 12 weeks (Nomura et al. 2014).

The human body possesses unique antioxidant redox hemostasis to maintain and stabilize the balance of oxidative molecules (Hamid et al. 2010). In the current study, Alcl3-induced decrease in hepatic and renal GSH content, indicating impairment in antioxidant defense system. These findings are matching with earlier study that showed that Alcl3 causes oxidative stress, which lowers the activity of GSH and inhibits the levels of antioxidant enzymes in various tissues (Kumar and Gill 2014). This study documented that the antioxidant enzymes activity was inhibited due to reduced GSH biosynthesis either from increased intracellular Al levels or excessive free radical production (Cheraghi and Roshanaei 2019). Supplementation of Alcl3-intoxicated rats with Feb could normalize the hepatic and renal activity of GSH indicating its ability to restore redox homeostasis. Previous research revealed that oral febuxostat (10 and 15 mg/kg/day, respectively) for 14 days beginning 7 days before cisplatin injection (Fahmi et al. 2016).

Our findings demonstrated that the Alcl3-treated group, Feb 10 mg/kg treatment significantly (*p* 0.05) lowered AST, ALT, and ALP. However, the aforementioned parameters might be returned to normal values with treatment with Feb 15 mg/kg. Exposure to Alcl3-induced disruption in hepatic and renal membranes leading to subsequent leakage of these biomarkers into the systemic circulation. Our findings concur with those of an earlier study that found that higher hepatic and kidney function indices are considered the chief symptoms of Alcl3 in a dose of (34 mg/kg which equal to 1/25 LD 50), induced hepatotoxicity and nephrotoxicity (El-Demerdash et al. 2022). Pretreatment with Feb significantly reduced Alcl3-induced hepatotoxicity and nephrotoxicity, as evidenced by reducing serum hepatic and kidney values. These results are consistent with a prior study that documented the ameliorative role of Feb during exposure to xenobiotics (Battelli et al. 2018).

Hepatotoxicity is reflected by significant alteration in lipid metabolism. Our findings revealed that administration of Alcl3 orally for 2 months caused a marked increment in the values of total lipids, triglycerides, and total cholesterol. As previously stated, Alcl3 administration leads to elevated MDA-induced disruption in membrane integrity causing significant impairment in lipid metabolism. Alterations in enzyme activities are considered the main factors in triggering hypertriglyceridemia and hypercholesterolemia and those results were in line with (Newairy et al. 2009). On the other hand, administration of Feb (15 mg/kg) could restore the forementioned parameters and our results were agreed (Heikal et al. 2019) that reported that Feb showed strong anti-inflammatory and antioxidant properties by lowering serum levels of proinflammatory cytokines and lipid peroxidation index and increasing antioxidant enzyme activity in high-fat diet rabbits.

Hepatotoxicity is thought to be indicated by abnormal protein metabolism. The administration of Alcl3 in the current study caused a marked reduction in the levels of albumin and total protein. This finding is in agreement with (Al-Eisa et al. 2017), administration of aluminium chloride in adose 30 mg/kg every other day intraperitoneally for 8 weeks revealed the decrease in total protein and albumin in Alcl3-treated animals might be related to alterations in the synthesis and/or metabolism of protein. On the other hand, administration of Feb (10 and 15 mg/kg) to Alcl3 intoxicated rats showed normalization in the levels of albumin and total protein indicating the occurrence of conformational changes in the albumin and protein levels and those findings are matched with prior study (Maresh et al. 2020).

The master regulator of several antioxidant enzymes, nuclear factor-erythroid 2-related factor 2 (Nrf2) modifies cellular redox equilibrium and detects the presence of oxidative stress. This is accomplished by increasing the activity of enzymes involved in antioxidant defense, including ferritin, glutathione reductase, glutathione peroxidase, heme oxygenase-1 (HO-1), and superoxide dismutase (SOD) (Younis et al. 2021). This redox sensitive transcription factor is also present in the cells to protect it from oxidative stress (Kensler et al. 2007). All tissues have constitutively high amounts of Nrf2, which might fluctuate depending on how much detoxification an organ needs to perform (Shelton and Jaiswal 2013). When exposed to oxidative stress, Nrf2 dissociates from Keap1 and moves to the nucleus where it binds with the antioxidant-response element (ARE) to control the transcription of antioxidant genes (Lewis et al. 2010). Nrf2 is kept in the cytosol by attaching to several proteins, including its cytosolic inhibitor, Kelch like-ECH-associated protein 1 (Keap1) (Wei et al. 2011).

According to this study, rats exposed to Alcl3 had significantly lower levels of Nrf2 and MNK expression in their liver and kidney tissues than did control rats (*P* 0.05). However, compared to rats merely exposed to Alcl3, rats treated with Feb (10 mg/kg) after being exposed to Alcl3 had considerably higher expression levels of Nrf2 and MNK in their liver and kidney tissues. Moreover, Alcl3-exposed rats treated with Feb (15 mg/kg) had considerably higher expression levels of the Nrf2 gene in their liver and kidney tissues than Alcl3-exposed rats treated with Feb (10 mg/kg), but not of the MNK gene. Our findings are consistent with thoseof (Chen et al. 2021) who found a connection between Nrf2 dysregulation and Alcl3 toxicity which induced by oral administration of 175 mg/kg of AlCl3 for 25 days. This suggests that increased oxidative stress created by the Alcl3 environment signals the cell to produce more Nrf2, but despite increased production, it fails to reach the nucleus to augment the transcription machinery. And thus may explain the mechanisms of action of AL in our body that produces free radicals via, increases the ability to uptake iron; thus, oxidative stress may contribute the Alcl3 toxicity (Al-Kahtani and Morsy 2019).

One of our most interesting results is that the oral administration of Feb (10 mg/kg and 15 mg/kg b.w.) concurrent with Alcl3 upregulated the hepatic and renal levels of the Nrf2 gene. This would imply that one of the Feb’s primary defense mechanisms against Alcl3-induced toxicity is activation of Nrf2. Our results are consistent with those of (Omizo et al. 2020), who hypothesized that Feb was dissolved in drinking at a concentration of 0.03 mg/L for 8 weeks might facilitate the nuclear translocation of Nrf2, that potentiate the renal antioxidant capacity. Additionally, previous studies showed that elevated Nrf2 expression was associated with reduced inflammation in hepatic ischemia–reperfusion injury; therefore, we could conclude that Nrf2 mediates antioxidant, the anti-inflammatory, and antiapoptotic effects (Zhong et al. 2013).

Recent research also revealed that the activation of numerous signaling pathways, including PI3Kphosphatidylinositol 3-kinase/Akt, protein kinase B PKC protein kinase C, and MNK mitogen-activated protein kinase, allows the release of Nrf2 from Keap1 and subsequent translocation for the induction of diverse antioxidant and detoxifying enzyme expressions (Martin et al. 2004). MAP kinase-interacting serine/threonine kinase (MNK) was discovered as a subfamily of murine serine/threonine kinase by screening a mouse embryo library with a novel ERK-interacting clone as a probe (Guo et al. 2017). The MNK pathway controls a number of cellular processes; cancer, neurodegeneration, and inflammation have all been linked to dysregulation of MAPK kinase pathways (Hassan and Kadry 2021a). JNK, MNK P38 and extracellular signal-regulated kinase (ERK) are members of the MAPK family. Its activation has recently been claimed to have a crucial role in the pathophysiology of hepatic encephalopthy (Khalil et al. 2022).

Our current study revealed that Alcl3-induced a significant downregulation in hepatic and renal MNK (ERK). This could imply that MNK (ERK) activation is one of the Feb’s key protective mechanisms against Alcl3-induced toxicity. Furthermore, the effect of Feb on MNK (ERK) expression in the liver and kidneys of treated rats were correlated with the activities of antioxidant enzymes in this study. Activating MNK (ERK) can prevent oxidative stress-induced cell apoptosis and thus consistent with these findings, in our study, co-treatment with Alcl3 and Feb (10 and 15 mg/kg) cause activation of ERK and alleviated ROS generation, which reveals that MNK, Alcl3 and oxidative stress may influence each other. Our results are consistent with (Khan et al. 2017) who demonstrated that Feb pretreatment activate the ERK1/2 pathway and suppressing the p38/JNK/NF-κBp65/T TNF-α pathway and thus reduced the heart dysfunction brought on by reperfusion injury.

The basic mechanism of carcinogenesis is oxidative stress (OS), and methylation of its associated genes may contribute to the development of cancer. A crucial part of several metabolic processes is played by the mitochondrial matrix enzyme that CRAT generates, which catalyzes the inter conversion of acetyl-CoA and acetyl carnitine. According to studies, CRAT not only regenerates free CoA but also buffers the mitochondrial acetyl-CoA pool, both of which have an impact on the actions of a number of oxidative enzymes. Insufficient CRAT activity may worsen number of metabolic disturbances and raise oxidative stress levels (Seiler et al. 2014).

When compared to control rats, the group of rats treated to Alcl3 showed a substantial upregulation in the expression levels of the Crat and Car3 genes in the liver and kidney tissues. However, compared to rats exposed to Alcl3 alone, animals treated with Feb (10 mg/kg) showed significantly lower levels of Crat and Car3 expression in the liver and kidney tissues. Additionally, rats exposed to Alcl3 who were given Feb (15 mg/kg) had significantly lower Crat and Car3 expression levels in their liver and kidneys than rats exposed to Alcl3 who were given Feb (10 mg/kg).

So, this will decrease oxidative stress levels and enhance different metabolic processes. Our results are consistent with Gao et al. 2016.

Carbonic anhydrase (car3) is a family of metalloenzymes, and its active site contains a zinc ion that has an important role in carcinogenesis. The primary job of car3 is to catalyze the reversible hydrolysis of carbon dioxide into bicarbonate. Car3 takes involvement in the transport of carbon dioxide, calcification, and photosynthesis. In mammals, car3 participates in the metabolism-related synthesis of glycogen, urea, and lipids and controls ion transport, pH value, and water homeostasis (Bolt et al. 2005). Furthermore, the ability of Feb to reduce the levels of crat and car 3 (procarcinogenic genes) in the hepatic and renal tissue of the Alcl3-feb groups supported this assumption. These findings are in parallel with Szollosi et al. 2020 who showed that car3 enhances the capacity of hepatocellular carcinoma cells to invade through the FAK signaling pathway. In this study it was observed that the Alcl3 group showed over expression of car3 when compared to the control group. While, other researchers have made use of car3 inhibitors, such as acetazomide, methazolamide, ethoxzolamide, dichlorophenamide, dorzolamide, and brinzolamide. According to certain research, both in vivo and in vitro cancer cell growth, proliferation, migration, and colony formation may be considerably decreased by car3 inhibitors (Karakuş et al. 2018). This assumption is in harmony with Prat et al. 1985. Who revealed that a link between car3 and tumor metastasis.

According to the results of molecular docking studies, Feb showed strong binding capacity with the core targets of inflammatory cascades especially, COX-1, MAPK-p38 and NF-κB.

## Conclusion

Our current study used two dose levels of febuxostat (10 and 15 mg/kg/day), where, the latter dose appears the most effective. Based on our findings, we can conclusively report that Feb can avert Alcl3-induced hepatotoxicity and nephrotoxicity due to its greatest activity to scavenge ROS, triggering the activity of antioxidant enzymes, and inhibiting the inflammatory cascade and apoptosis. As a result, Feb is crucial for the development of cutting-edge therapeutic approaches that aimed to counteract Alcl3-induced hepatotoxicity and nephrotoxicity.

## Data Availability

All data are available upon request.

## Abbreviations

Feb: Febuxostat
Al: Aluminum
Crat: Carnitine o-acetyltransferase
Car3: Carbonic anhydrase
MNK: MAP kinase-interacting serine/threonine kinase
MDA: Malondialdehyde
GSH: Glutathione
Nrf2: Nuclear factor erythroid 2-related factor 2
TNF-α: Tumor necrosis factor-alpha
COX-1: Cyclooxygenase-1
NIK: NF-kappa-B-inducing kinase
MAPK-p38: Mitogen-activated protein kinases-p38
Alcl3: Aluminum chloride
ROS: Reactive oxygen species
PBS: Phosphate-buffered saline
NRC: National Research Centre
ANOVA: One-way analysis of variance
LPO: Lipid peroxidation
OS: Oxidative stress
ARE: Antioxidant-response element
HO-1: Heme oxygenase-1
SOD: Superoxide dismutase (SOD)
DEPC: Diethylpyrocarbonate
GLM: General liner models
AST: Alanine amino transferase
ALT: Aspartate amino transferase
ALP: Alkaline phosphatase
BBB: Blood brain barrier
